# BK_Ca_ Channel Inhibition by Peripheral Nerve Injury Is Restored by the Xanthine Derivative KMUP-1 in Dorsal Root Ganglia

**DOI:** 10.3390/cells10040949

**Published:** 2021-04-20

**Authors:** Kuang-I Cheng, Kan-Ting Yang, Chien-Lun Kung, Yu-Chi Cheng, Jwu-Lai Yeh, Zen-Kong Dai, Bin-Nan Wu

**Affiliations:** 1 Department of Anesthesiology, School of Medicine, College of Medicine, Kaohsiung Medical University, Kaohsiung 807, Taiwan; kuaich@kmu.edu.tw; 2 Department of Anesthesiology, Kaohsiung Medical University Hospital, Kaohsiung 807, Taiwan; 3 Department of Pharmacy, Kaohsiung Chang Gung Memorial Hospital, Kaohsiung 833, Taiwan; r3809922@cgmh.org.tw; 4 Department of Pharmacology, Graduate Institute of Medicine, College of Medicine, Drug Development and Value Creation Research Center, Kaohsiung Medical University, Kaohsiung 807, Taiwan; nmm20013@gmail.com (C.-L.K.); u9251055@gmail.com (Y.-C.C.); jwulai@kmu.edu.tw (J.-L.Y.); 5 Department of Medical Research, Kaohsiung Medical University Hospital, Kaohsiung 807, Taiwan; 6 Department of Pediatrics, School of Medicine, College of Medicine, Kaohsiung Medical University, Kaohsiung 807, Taiwan; 7 Department of Pediatrics, Division of Pediatric Cardiology and Pulmonology, Kaohsiung Medical University Hospital, Kaohsiung 807, Taiwan

**Keywords:** KMUP-1, chronic constriction injury, dorsal root ganglion, perforated patch-clamp, BK_Ca_ currents, immunofluorescent staining

## Abstract

This study explored whether KMUP-1 improved chronic constriction injury (CCI)-induced BK_Ca_ current inhibition in dorsal root ganglion (DRG) neurons. Rats were randomly assigned to four groups: sham, sham + KMUP-1, CCI, and CCI + KMUP-1 (5 mg/kg/day, i.p.). DRG neuronal cells (L4–L6) were isolated on day 7 after CCI surgery. Perforated patch-clamp and inside-out recordings were used to monitor BK_Ca_ currents and channel activities, respectively, in the DRG neurons. Additionally, DRG neurons were immunostained with anti-NeuN, anti-NF200 and anti-BK_Ca_. Real-time PCR was used to measure BK_Ca_ mRNA levels. In perforated patch-clamp recordings, CCI-mediated nerve injury inhibited BK_Ca_ currents in DRG neurons compared with the sham group, whereas KMUP-1 prevented this effect. CCI also decreased BK_Ca_ channel activity, which was recovered by KMUP-1 administration. Immunofluorescent staining further demonstrated that CCI reduced BK_Ca_-channel proteins, and KMUP-1 reversed this. KMUP-1 also changed CCI-reduced BK_Ca_ mRNA levels. KMUP-1 prevented CCI-induced neuropathic pain and BK_Ca_ current inhibition in a peripheral nerve injury model, suggesting that KMUP-1 could be a potential agent for controlling neuropathic pain.

## 1. Introduction

Nerve injury-induced neuropathic pain is persistent, and it may sometimes never be completely relieved [1]. Hyperalgesia and allodynia associated with neuropathic pain are the hallmarks of peripheral nerve injury. Neuropathic pain is a complex disorder that leads to chronic illness and adversely affects the patient’s quality of life. Therapeutic intervention using various pharmacological agents remains the most desirable option for chronic neuropathic pain, but the results are not overwhelmingly successful, and not all patients achieve sufficient pain relief [2,3,4]. There is a considerable unmet need for improved treatments for these patients [5]. So far, neuropathic pain management is oriented toward symptom-directed therapy instead of elucidating the predisposing mechanisms responsible for the pain. Our understanding of the pathophysiology that leads to neuropathic pain is incomplete [2,3,6]. Comprehensive studies of neuropathic pain are making progress in the search for better alternatives, however. The chronic constriction injury (CCI) model helps understand nociception and chronic pain pathogenesis [7,8].

Damage to the peripheral nervous system produces long-lasting hyperexcitability of sensory neurons in diverse species [9]. In mammals, injury affecting the axons or somata of sensory neurons having their somata in dorsal root ganglion (DRG) often causes hyperexcitability, leading to spontaneous firing, neuropathic pain, and paresthesias [10]. Although electrophysiological mechanisms contributing to the expression of hyperexcitability in DRG neuronal somata after an injury to the peripheral nervous system have been studied intensively, less is known about the signals that induce and maintain this hyperexcitability [11].

There are three types of Ca^2+^-activated K^+^ channels based on their conductance: large conductance (BK_Ca_), intermediate conductance (IK), and small conductance (SK) channels [12]. All three channels are expressed in rat DRG neurons [13]. BK_Ca_ channels are widely distributed throughout the central nervous system and play a critical role in regulating neuronal action potential duration and firing frequency, along with presynaptic neurotransmitter release [14]. The BK_Ca_ channel can be activated by a depolarizing voltage and increases in intracellular Ca^2+^. Some reports demonstrated that spinal nerve injury produces a decrease in BK_Ca_ channel expression and activity in the DRG neurons. Blocking those BK_Ca_ channels at the spinal level can mimic neuropathic pain in rats [13,15]. However, the functional role of BK_Ca_ channels in DRG neurons has not been extensively investigated.

The xanthine derivative KMUP-1 (7-[2-[4-(2-chlorobenzene)piperazinyl]ethyl]-1,3-dimethylxanthine) is known to increase protein kinase A (PKA) and PKG and activate K^+^ channels, resulting in smooth muscle relaxation [16,17,18]. It can decrease cardiac hypertrophy via the NO/cGMP/PKG pathway [19]. KMUP-1 was further shown to prevent pulmonary arterial hypertension through Rho kinase inhibition and K^+^-channel activation [20,21]. KMUP-1 activates BK_Ca_ (large-conductance Ca^2+^-activated K^+^) channels [22] and inhibits L-type Ca^2+^ channels [23] in rat basilar arteries. Apart from BK_Ca_ channel activation, KMUP-1 preferentially suppresses late Na^+^ current (I_Na_) in pituitary GH3 cells [24]. Moreover, KMUP-1 has been demonstrated to control cerebral vasospasm after subarachnoid hemorrhage due to its K^+^-channel opening activities [25]. Emerging evidence shows that opening the K^+^-channel is associated with antinociception [26]. Accordingly, K^+^ channels could be a perfect target for developing antinociceptive drugs. As a K^+^-channel opener, KMUP-1 has been demonstrated to reduce hyperalgesia and inflammation in a bilateral CCI model due to the suppression of mitogen-activated protein kinase (MAPK)-dependent nuclear factor-κB (NFκB) activation [27]. However, the role of KMUP-1 on peripheral nerve injury-induced BK_Ca_ channel changes in DRG neurons remains unexplored. This study’s main objective was to investigate whether KMUP-1 could modulate BK_Ca_ channels in DRG neuronal cells after CCI injury.

## 2. Materials and Methods

### 2.1. CCI Animal Model

Male Sprague Dawley rats weighing 250–300 g were purchased from the National Laboratory Animal Breeding and Research Center (Taipei, Taiwan) and housed under constant temperature and controlled illumination. Food and water were available ad libitum. All procedures were approved by the Animal Care and Use Committee (IACUC #105112) at Kaohsiung Medical University. They complied with the Guide for the Care and Use of Laboratory Animals published by the US National Institutes of Health. Bilateral CCI of the sciatic nerves was performed as described previously [8,27,28]. This animal model is reproducible for exploring possible therapeutic interventions in spontaneous or stimulus-evoked pain [28,29]. Rats were randomly divided into four groups: (1) sham; (2) sham + KMUP-1; (3) CCI; (4) CCI + KMUP-1. Rats were injected intraperitoneally (i.p.) with KMUP-1 (5 mg/kg/day) once daily for both treatment groups (CCI and sham-operated, *n* = 6), starting on the next day after surgery. We used the same dose of KMUP-1 as in our previous publication [27]. The experimental endpoint was a sacrifice on day 7, at the peak of pain behavior induced by CCI [27,30].

### 2.2. Isolation of DRG Cells and Cell Culture

DRG neurons were dissociated from L4 to L6 ganglia taken from rats using conventional methods [31,32]. In brief, the excised ganglion was minced using microdissection scissors. The DRG fragments were transferred into 5 mL of the cold phosphate-buffered saline (PBS) containing (in mM): 138 NaCl, 3 KCl, 10 Na_2_HPO_4_, 2 NaH_2_PO_4_, 5 glucose, 0.1 CaCl_2_, and 0.1 MgSO_4_ (pH 7.4) with collagenase type IA (0.15 mg/mL) and then incubated for 30 min at 37 °C. The DRG fragments were removed, rinsed five to six times in the PBS solution, and put into the PBS solution (5 mL) containing DNase (0.2 mg/mL, Sigma-Aldrich, St. Louis, MO, USA) to prevent possible toxicity from DNA leaking from ruptured cells [32]. Individual neurons were dissociated by passing DRG fragments through a set of fire-polished glass pipettes. Dissociated DRG cells were then transferred to the polydimethylsiloxane (PDMS)-coated coverslips with fibronectin [31], and electrophysiological recordings were performed at room temperature ~6 h after dissociation. For immunofluorescence, DRG neuronal cells were cultured in DMEM supplemented with 2 mM L-glutamine, 10% fetal bovine serum (GIBCO, Carlsbad, CA, USA), and 1% penicillin/streptomycin (GIBCO), placed in an incubator (37 °C) and saturated with water vapor (5% CO_2_, 95% air) after a two-day incubation.

### 2.3. Perforated and Inside-Out Patch-Clamp Electrophysiology

Whole-cell BK_Ca_ currents were measured using the perforated patch-clamp configuration as previously described [22]. In brief, DRG cells were placed in a recording dish and perfused with a bathing solution contained (in mM): 140 NaCl, 5 KCl, 1.8 CaCl_2_, 1 MgCl_2_, 10 HEPES, and 10 glucose (pH 7.4, NaOH). In comparison, the pipette solution contained (in mM): 140 KCl, 0.1 CaCl_2_, 1 MgCl_2_, 1 EGTA, and 10 HEPES (pH 7.2, KOH). Amphotericin B (200 μg/mL) was included in the perforated patch-clamp recordings in the pipette solution to prevent dialyzing intracellular calcium. A recording electrode was pulled from borosilicate glass (resistance: 3–5 MΩ). The tip was covered with sticky wax and backfilled with pipette solution. The electrode was gently lowered onto a DRG cell. Negative pressure was briefly applied, and a gigaohm seal was obtained, and then membrane perforation was achieved ~10 min. Cells were subsequently voltage-clamped (−80 mV). Membrane currents were recorded on a MultiClamp 700B amplifier (Molecular Devices, Sunnyvale, CA, USA), filtered at 2 kHz using a low-pass Bessel filter, digitized at 5 kHz, and stored on a computer for subsequent analysis with Clampfit 10.2. A 1 M NaCl-agar salt bridge between the bath and the Ag-AgCl reference electrode was used to minimize offset potentials. All electrical recordings were performed at room temperature.

For employing the inside-out patch-clamp configuration to monitor single-channel BK_Ca_ activity [25], and recording pipettes were backfilled with a solution containing (in mM): 140 KCl, 1.8 CaCl_2_, 1 MgCl_2_, 10 HEPES, and 10 glucose (pH 7.2, KOH). The bath solution contained (in mM) 140 KCl, 1.8 CaCl_2_, 1 MgCl_2_, 10 HEPES, 5 EGTA, and 10 glucose (pH 7.2, KOH). The Max Chelator Sliders software (ver. 2.5, C. Patton, Stanford University, Pacific Grove, CA, USA) calculated appropriate free Ca^2+^ concentration. The BK_Ca_ channel activity (NPo) was determined from continuous gap-free data by using Clampfit 10.2. The NPo was calculated from the following equation: NPo = (Σt_i_ × i)/T, where i is the number of channels open, t_i_ is the open time for each level i, and T is the total time of analysis.

Whole-cell voltage-clamp DRG cells (diameter:15–30 μm with a calibrated eyepiece reticule [33], capacitance: 12.6 ± 1.2 pF) for measuring membrane currents were equilibrated for 10 min before experimentation. Following equilibration, whole-cell BK_Ca_ currents were monitored. In general, the net current-voltage (I-V) relationship was determined at 5 min intervals by measuring the peak current at the end of a 300 ms pulse to voltages between −80 and +60 mV for BK_Ca_ currents. In the inside-out configuration, DRG cells were clamped at 0 mV to monitor the channel activity of BK_Ca_.

### 2.4. Immunofluorescent Staining

DRG neuronal cells cultured on glass coverslips were fixed with 10% neutral buffered formalin and permeabilized with 0.1% Triton X-100 in phosphate-buffered saline (PBS). After blocking with 3% bovine serum albumin in PBS, the coverslips were incubated with primary antibodies BK_Ca_ (Alomone Labs, Jerusalem, Israel) and NeuN, Cy3 Conjugate (neuronal marker, Sigma-Aldrich, St. Louis, MO) overnight at 4 °C. Next, the coverslips were incubated with Alexa Fluor 488 and 594 for 1 h at room temperature. Alexa Fluor 488 and 594 are green and red fluorescent dyes, respectively. Images were acquired by a Zeiss LSM700 equipped with Zen software (ver. 3.3, Oberkochen, Germany) to process the image. The relative fluorescence intensity was normalized to the sham group.

### 2.5. Quantitative Real-Time Polymerase Chain Reaction

Total RNA was extracted from each rat of DRG neuronal cells using the RNeasy Lipid Tissue Mini Kit (Qiagen, Ann Arbor, MI, USA). According to the manufacturer’s instructions, reverse transcription was executed with 1 μg total RNA using the Quantitect Reverse Transcription Kit (Qiagen). Real-time PCR with BKα subunit (KCNMA1) primers (Forward: 5′-CCGTCCACAGCAAATCGGCCA-3′; Reverse: 5′-CCATGTGGGTACTCATGGGCTTGG-3′) or actin (ACTB) primers (Forward: 5′-TGTCACCAACTGGGACGATA-3′; Reverse: 5′-GGGGTGTTGAAGGTCTCAAA-3′) was carried out using the Quantitect SYBR green PCR kit (Qiagen). Rat ACTB was used as the reference gene. PCR reaction was performed at 95 °C for 10 min, followed by 40 cycles of 95 °C for 15 sec and 60 °C for 1 min. Relative mRNA expression levels were normalized to rat actin. BK_Ca_ mRNA results were normalized to the sham group (% of sham). The 2^−ΔΔCt^ method calculated the gene expression, where the ΔCt is the difference in threshold cycle (Ct) between the target and reference genes.

### 2.6. Chemicals

Buffer reagents, collagenase type IA, l-glutamate, amphotericin B, paxilline, and trypsin were obtained from Sigma-Aldrich Chemical Co. (St. Louis, MO). All drugs and reagents were dissolved in distilled water unless otherwise stated. KMUP-1 was dissolved in 10% absolute alcohol, 10% propylene glycol, and 2% 1 N HCl at 10 mM. Serial dilutions were made in phosphate buffer solution, with the final solvent concentration < 0.01%.

### 2.7. Data Analysis and Statistics

Data are expressed as means ± SE, and n indicates the number of animals per group, each conducted in triplicate and the number of analyzed neuronal cells. One-way ANOVA analyzed differences between groups with the post-hoc test. For perforated whole-cell recording, repeated measures ANOVA compared values at a given voltage followed by the Tukey–Kramer test, while others used ANOVA followed by the Tukey–Kramer test. For all statistical analyses, *p* < 0.05 was considered statistically significant.

## 3. Results

### 3.1. DRG Neuronal Cells Express Functional BK_Ca_ Channels

**Figure 1:**
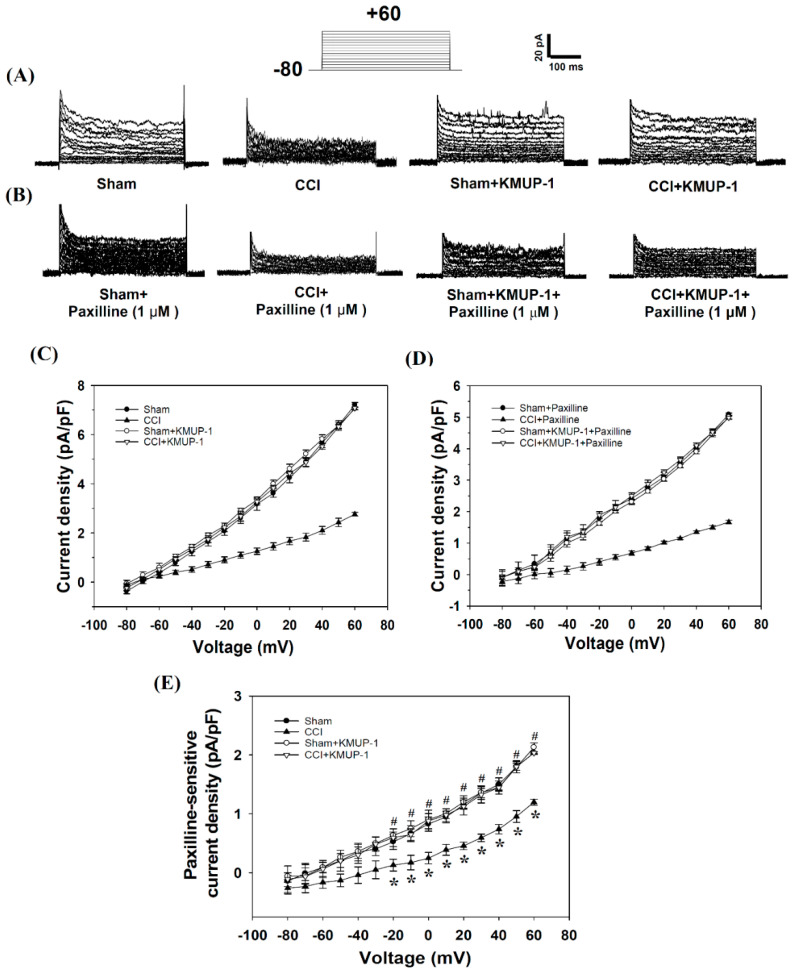
KMUP-1 prevented CCI-induced nerve injury decreases in BK_Ca_ currents in DRG neuronal cells. (**A**) Outward currents from sham, CCI, sham + KMUP-1, and CCI + KMUP-1 (5 mg/kg/day) groups. (**B**) Outward currents in the presence of the selective BK_Ca_ inhibitor paxilline (1 μM) from sham, CCI, sham + KMUP-1, and CCI + KMUP-1. Currents were elicited by a series of 10 mV depolarizing steps (–80 to 60 mV) from a holding potential of –80 mV. Current-voltage (I-V) curve of outward currents without (**C**) and with paxilline (**D**). (**E**) BK_Ca_ currents were defined as the difference between outward current recorded without and with paxilline. Data are means ± SE, *n* = 6, each conducted in triplicate. Repeated measures ANOVA followed by the Tukey–Kramer test. * *p* < 0.05 compared with the sham group; # *p* < 0.05 compared with the CCI group.

In voltage-clamp mode, perforated whole-cell recordings were used to assess the effect of KMUP-1 on the regulation of outward BK_Ca_ conductance in DRG neuronal cells. Openings of BK_Ca_ channels were identified based on the characteristic single-channel conductance and blocked by iberiotoxin or paxilline as previously described [22,34]. DRG cells were voltage-clamped at –80 mV, and continuously superfused with an isotonic physiological solution containing 1.8 mM Ca^2+^ ± KMUP-1 (0, 1, 3, 10 μM). KMUP-1 (0, 1, 3, 10 μM)-induced increases in outward currents were measured in a dose-dependent manner (data not shown). Some data can also be found in Figure 1. The current density at 60 mV was 7.21 ± 0.10, 9.12 ± 0.06, 12.20 ± 0.19, 14.70 ± 0.24 pA/pF, *n* = 10–15 cells, *p* < 0.05, and inhibited by 1 μM paxilline, a selective BK_Ca_ inhibitor (4.86 ± 0.06, 6.65 ± 0.22, 9.12 ± 0.12, 11.06 ± 0.23 pA/pF, *n* = 10–15 cells, *p* < 0.05). As noted, the paxilline-sensitive outward currents (2.34 ± 0.02, 2.45 ± 0.04, 3.04 ± 0.05, 3.77 ± 0.07 pA/pF) were identified as BK_Ca_ currents. Data suggested that the DRG neuronal cells did have BK_Ca_ channels in the cell membrane.

### 3.2. KMUP-1 Prevents CCI-Induced Inhibition of BK_Ca_-Channel Activity and Current

In our animal study, rats were separated into four groups as previously described in the Materials and Methods. Isolated DRG neuronal cells were used for the subsequent experiments. The cell capacitance (~12.6 pF) of DRG neurons was tiny, and thus the BK_Ca_ currents measured were small as well. From our results, we hypothesized that DRG might exist subpopulations of neuronal cells with low current density characteristics of BK_Ca_ channels. Our electrophysiological recordings had a technique limitation. We visualized the DRG cells with a calibrated eyepiece reticule but did not stain the cells with isolectin B4 (IB4) Alexa Flour 488 conjugate. IB4-binding is a positive marker for the subpopulation of small-diameter DRG neurons [35].

**Figure 2:**
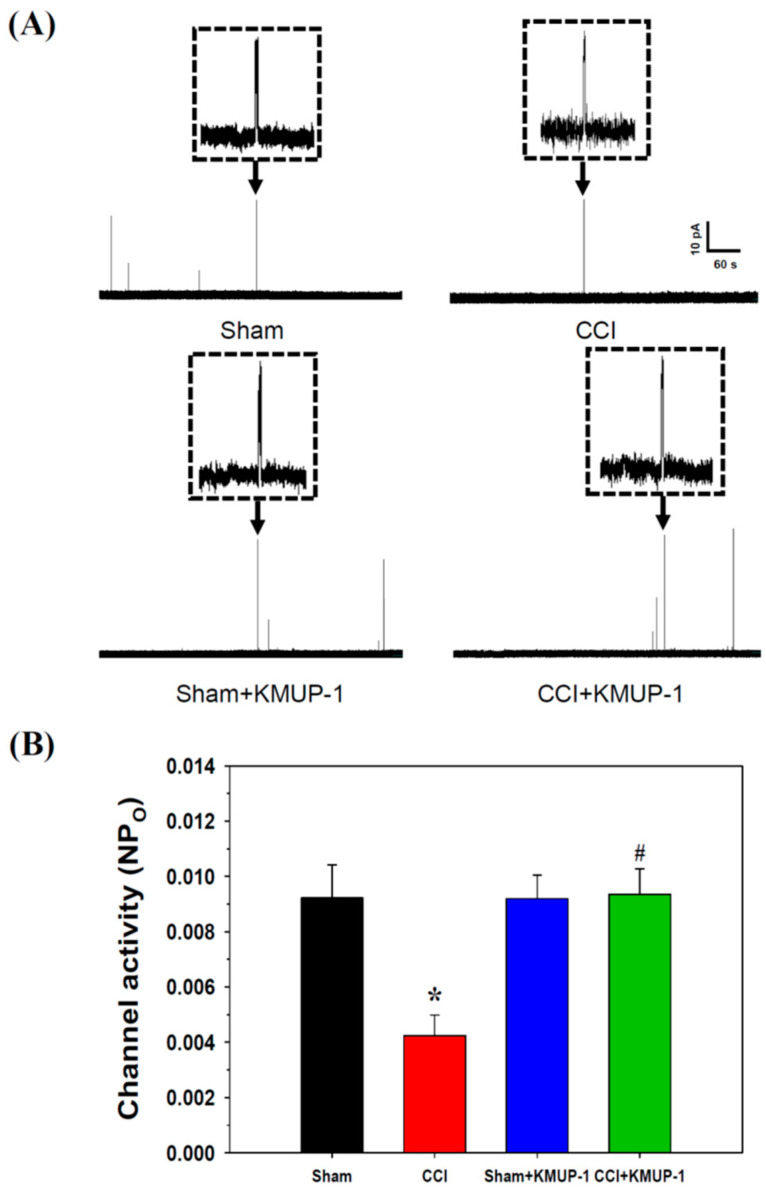
Effects of KMUP-1 on BK_Ca_ channel activity (NPo) in DRG neuronal cells using inside-out patches. (**A**) Original current recording from sham, CCI, sham + KMUP-1, and CCI + KMUP-1 (5 mg/kg/day), voltage-clamped at 0 mV. (**B**) Bar graph showing the NPo of BK_Ca_ channels from sham, CCI, sham + KMUP-1, and CCI + KMUP-1 groups. Data are means ± SE, *n* = 6, each conducted in triplicate. ANOVA followed by the Tukey–Kramer test. * *p* < 0.05 compared with the sham group; # *p* < 0.05 compared with the CCI group.

In perforated whole-cell recordings, BK_Ca_ currents were significantly reduced by CCI injuries on day 7 in rat DRG cells compared to the sham-operated rats. Intraperitoneal injection of KMUP-1 (5 mg/kg/day) restored CCI-attenuated BK_Ca_ currents without effects on the sham-operated rats (Figure 1). Thus KMUP-1 restoration of BK_Ca_ currents could address CCI-induced inflammation and pain behaviors. In inside-out recordings, CCI also attenuated the channel activity (NPo, 0.0092 ± 0.0012 to 0.0042 ± 0.0007, *p* < 0.05) of BK_Ca_, and this response was reversed by KMUP-1 administration (NPo, 0.0042 ± 0.0007 to 0.0095 ± 0.0010, *p* < 0.05) (Figure 2).

### 3.3. KMUP-1 Restores CCI-Inhibited BK_Ca_ Channel Proteins and mRNA Levels

**Figure 3:**
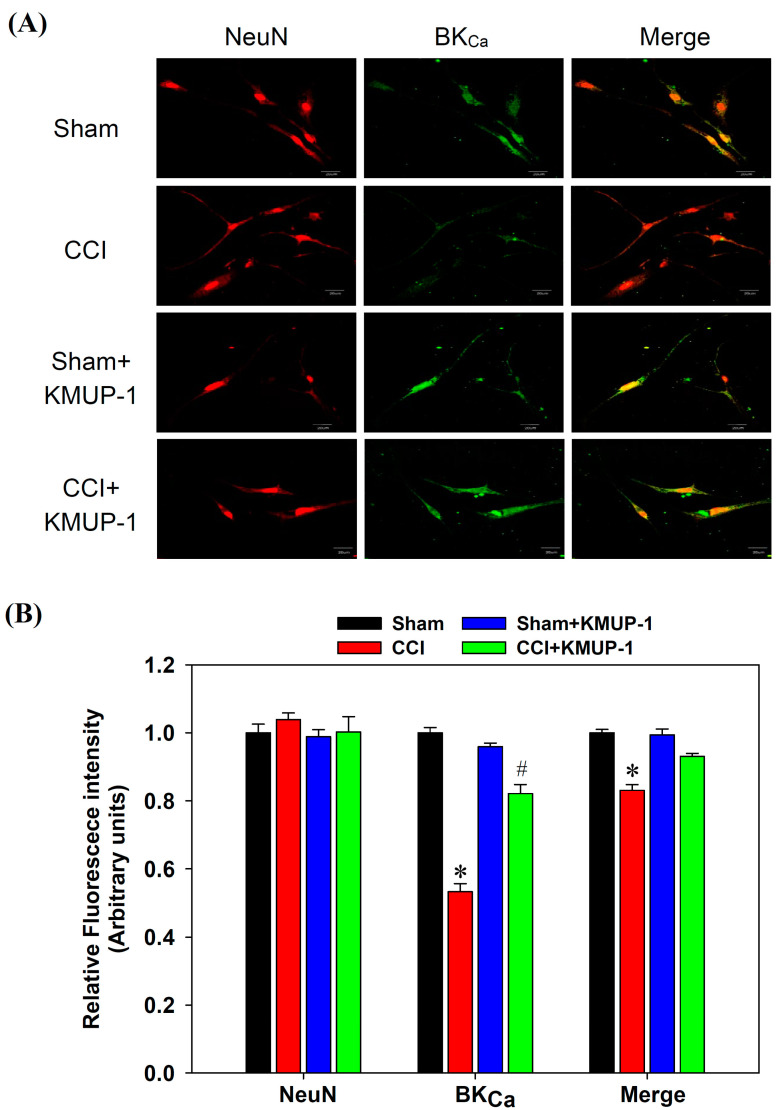
Confocal images show the BK_Ca_ channel immunoreactivities in DRG neuronal cells. (**A**) Images show the BK_Ca_ channel’s colocalization (green) with NeuN (red, neuronal marker) in DRG neurons from sham, CCI, sham + KMUP-1, and CCI + KMUP-1 (5 mg/kg/day) groups. (**B**) Bar graph showing the average relative fluorescence intensity. Data are means ± SE, *n* = 6, each conducted in triplicate. ANOVA followed by the Tukey–Kramer test. * *p* < 0.05 compared with the sham group; # *p* < 0.05 compared with the CCI group.

**Figure 4:**
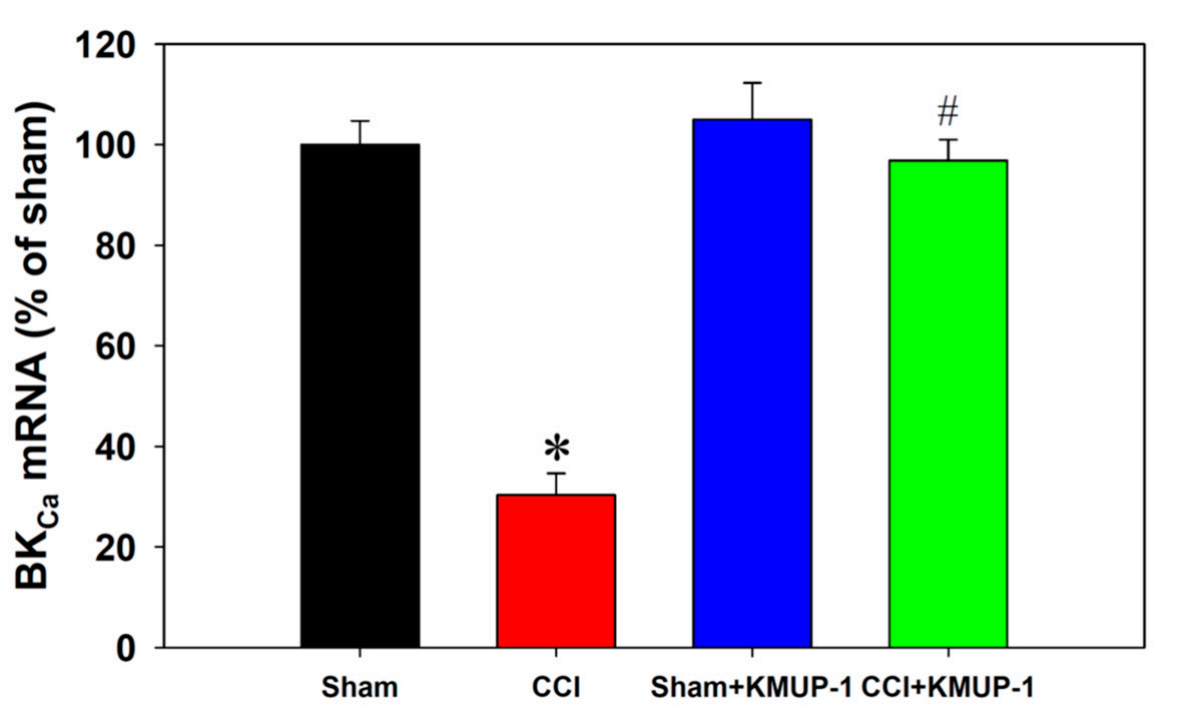
Quantification of the mRNA levels of BK_Ca_ channels in DRG neuronal cells. The bar graph shows BK_Ca_ mRNA expression from sham, CCI, sham + KMUP-1, and CCI + KMUP-1 (5 mg/kg/day) groups. Data are means ± SE, *n* = 6, each conducted in triplicate. ANOVA followed by the Tukey–Kramer test. * *p* < 0.05 compared with the sham group; # *p* < 0.05 compared with the CCI group.

To expose BK_Ca_ protein inhibition following CCI on day 7 in DRG neurons, we performed double immunofluorescent staining for measuring BK_Ca_ proteins and NeuN (a neuronal marker). The BK_Ca_ proteins showed a dramatic decrease in the CCI group, and this effect was reversed by the administration of KMUP-1 (5 mg/kg/day, Figure 3). Moreover, CCI also markedly reduced the mRNA levels of BK_Ca_ channels using real-time PCR, and this effect was prevented by KMUP-1 treatment as well (Figure 4).

## 4. Discussion

In this study, the administration of KMUP-1 restored CCI-mediated BK_Ca_ current inhibition in DRG neurons. CCI group decreased BK_Ca_ channel activity, an effect which KMUP-1 reversed. Immunofluorescence staining further demonstrated that CCI-induced nerve injury reduced BK_Ca_ channel expression, and the KMUP-1-treated group also showed improvement in these effects. KMUP-1 also changed CCI-reduced BK_Ca_ mRNA levels. Thus, KMUP-1 could be a potential therapeutic agent for preventing nerve injury and associated neuropathic pain due to the BK_Ca_ channel inhibition.

Some standard animal models were used to evaluate neuropathic pain, including spinal nerve ligation (SNL), spared nerve injury (SNI), and CCI [35]. In general, CCI is the best model for assessing peripheral nerve injury-induced neuropathic pain. CCI-induced neuropathic pain rat model, which closely mimics peripheral mononeuropathy in clinics [8,30]. SNL and SNI have also preferred animal protocols by which to simulate human neuropathic pain. Both CCI and SNL are favored to investigate the effect of nerve injury on K^+^ channel function [36].

Increased excitability of DRG neurons and nociceptive nerve endings alters axonal conduction and increases neurotransmitter secretion from primary afferent nerve terminals in the spinal dorsal horn. DRG neurons play an essential role in generating ectopic activity after nerve injury [36]. A previous report demonstrated that nerve injury reduces BK_Ca_ channel expression in the DRG but not in the spinal cord [13]. DRG neuron depolarization could promote hyperexcitability, and it is possible in relation to K^+^ channels other than BK_Ca_. However, this membrane depolarization could also inactivate voltage-dependent Na^+^ channels responsible for action potential trigger and reduce DRG neurons’ excitability. BK_Ca_ channels modulate neuronal excitability by shorting the action potential duration, accelerating after-hyperpolarization, enhancing the repolarization, and mediating spike-frequency adaption [14,15]. BK_Ca_ channels act as a functional brake that prevents neuronal hyperexcitability. The loss of BK_Ca_ channels in DRG neurons may be an essential step toward developing neuropathic pain after nerve injury [37]. One previous report demonstrated that nerve injury reduces BK_Ca_ channel activity in the DRG neurons [15]. Our CCI rat-induced BK_Ca_ current and channel activity inhibition are consistent with their findings. This study also observed that CCI-induced nerve injuries decrease BK_Ca_ mRNA expression and immunoreactivity in the DRG neuronal cells. Thus, we suggest that DRG neuron excitability is augmented in this rat model following CCI surgery.

It is generally agreed that small and medium DRG neurons are likely to convey pain and temperature information. In contrast, large DRG neurons are more likely to transfer mechanoreceptive details [36,38]. In our recent study, KMUP-1 was observed to have a strong analgesic effect in thermal hyperalgesia and had a modest impact on mechanical allodynia, suggesting that it could be more sensitive on Aδ and C fibers than on Aβ fibers [27]. Previous reports showed that L5 and L6 spinal nerve ligation suppressed BK_Ca_ channel expression and activity in the DRG neurons [13,15]. This study confirmed that CCI-induced nerve injuries also inhibit BK_Ca_ current and channel activity in DRG cells. Therefore, we suggested that improvement of both BK_Ca_ expression and function by KMUP-1 is involved in protecting CCI -induced nerve injury from the rats. KMUP-1 has been shown to have BK_Ca_-channel opening activity in vascular smooth muscle cells [22,25], and it also protects against CCI-induced neuroinflammation and pain behaviors (thermal hyperalgesia and mechanical allodynia). Accordingly, we consider that KMUP-1′s anti-neuropathic pain effects would partly involve this channel’s modulation. This study confirmed that KMUP-1 could prevent CCI-inhibited BK_Ca_ current and channel activity in rat DRG neurons using perforated whole-cell recordings. Since we cannot exclude the possibility of Na^+^, Ca^2+^, and other channels involved, we suggest that the action mechanism of KMUP-1 protection against neuropathic pain may be partly due to its restoration of CCI-inhibited BK_Ca_ channels.

In summary, our findings revealed that CCI-induced peripheral nerve injury diminishes the activity of BK_Ca_ channels in DRG neurons. KMUP-1 improved the CCI-mediated BK_Ca_ channel inhibition and reduced BK_Ca_ mRNA and immunoreactivity, which may benefit neuropathic pain in a rat model following peripheral nerve injury. Our results provide evidence that KMUP-1 prevents CCI-induced pain behaviors and BK_Ca_ channel inhibition, suggesting it may be a good candidate for managing neuropathic pain.

## Data Availability

The data that support the findings of this study are available from the corresponding author upon request.
